# Cytoprotective and antioxidant effects of *Echium amoenum* anthocyanin-rich extract in human endothelial cells (HUVECs)

**Published:** 2015

**Authors:** Leila Safaeian, Shaghayegh Haghjoo Javanmard, Mustafa Ghanadian, Sima Seifabadi

**Affiliations:** 1 *Department of Pharmacology and Toxicology and Isfahan Pharmaceutical Sciences Research Center, School of Pharmacy and Pharmaceutical Sciences, Isfahan University of Medical Sciences, Isfahan, Iran*; 2 *Applied Physiology Research Center, Isfahan University of Medical Sciences, Isfahan, Iran*; 3 *Department of Pharmacognosy, Isfahan Pharmaceutical Sciences Research Center, School of Pharmacy and Pharmaceutical Sciences, Isfahan University of Medical Sciences, Isfahan, Iran*

**Keywords:** *Echium amoenum*, *HUVECs*, *Oxidative stress*, *Antioxidant*

## Abstract

**Objective:**

* Echium amoenum *Fisch. & C.A. Mey. is used for the treatment of various diseases in traditional medicine. This plant is a major source of anthocyanins with beneficial cardiovascular properties such as anti-atherosclerotic and antihypertensive effects. In the present study, the protective and antioxidant effects of anthocyanin-rich *E. amoenum* extract were evaluated on human vascular endothelial cells (HUVECs) under oxidative stress.

**Materials and Methods:**

Cell viability and oxidative status were assessed on H_2_O_2_-induced oxidative stress (0.5 mM H_2_O_2 _for 2 h) in HUVECs pretreated by anthocyanin-rich extract from the petals of *E. amoenum *(25-1000 µg/ml). Cytoprotective effect of the extract was evaluated by 3-(4,5-Dimethylthiazol-2-yl)-2,5-diphenyltetrazolium bromide (MTT) assay. The hydroperoxides concentration and ferric reducing antioxidant power (FRAP) were assessed in intra- and extra-cellular fluid of pretreated cells.

**Results:**

Pretreatment of HUVECs with *E. amoenum* extract at the concentrations of 100-1000 µg/ml reduced the cell death resulted from the exposure to H_2_O_2 _in a concentration-dependent manner. *E. amoenum* extract decreased hydroperoxides concentration and increased FRAP value in both intra- and extra-cellular fluid at different concentration ranges. Moreover, it did not show cytotoxic effects at the concentration range of 25-1000 µg/ml.

**Conclusion:**

These results suggest antioxidant and protective effect of anthocyanin-rich extract of the petals of *E. amoenum* against H_2_O_2_-induced oxidative stress in HUVECs. However, further investigations are needed for understanding the detailed mechanisms of cytoprotective effects of this traditional herbal medicine.

## Introduction

Cardiovascular diseases (CVDs) are the leading cause of death in the world. In 2008, an estimated 17.3 million people died from CVDs which accounts for 30% of all deaths worldwide. It is estimated that 23.3 million deaths will occur as a result of CVDs by 2030. Physical inactivity, high blood pressure, obesity, and unhealthy diet are known as the main factors contributing to CVDs (WHO, 2009 ▶).

Oxidative stress, as the major reason of endothelial dysfunction, has a key role in the pathogenesis and in the development of CVDs including atherosclerosis, angina, stroke, and heart failure (Vogiatzi et al., 2009 ▶). Oxidative stress is resulted from excessive production of reactive oxygen species (ROS). They reduce antioxidant capacity of plasma and disrupt nitric oxide function and can involve multiple organs including the heart, the kidneys, vascular and nervous systems. In the vascular system, ROS directly inactivate the NO and consequently alter the function of the enzymes involved in vascular homeostasis (Schulz et al., 2004 ▶). Furthermore, oxidative stress increases oxidation of low density lipoprotein (LDL) and might lead to atherosclerosis (Peluso et al., 2012 ▶). Additionally, oxidative stress can induce apoptosis of endothelial and myocardial cells following different stimulus including ischemia, hypoxia, reperfusion, and inflammation and therefore plays an important role in endothelial dysfunction, inflammation, and hypertrophy (Touyz and Briones, 2011 ▶).

Previous studies have shown that antioxidants could improve endothelial dysfunction in various diseases such as atherosclerosis, hypertension, diabetes, and dyslipidemia (Carr and Frei, 2000 ▶). Antioxidants have an important role in the prevention of atherosclerosis by lowering the oxidation of LDL (Diaz et al., 1997 ▶). Furthermore, herbal antioxidants including polyphenols have been efficient in the prevention of endothelial cell apoptosis following exposure to oxidizing agents such as hydrogen peroxide (Touyz and Briones, 2011 ▶). Different studies have revealed the role of polyphenols in the improvement of endothelium-dependent vasodilatory responses and modulation of homeostatic endothelium-leukocyte interactions. Moreover, it has been shown that they could balance pro- and anti-thrombotic properties (Praticò, 2005 ▶).

*E. amoenum* (Boraginaceae) is known as a traditional remedy and possesses antioxidant, analgesic, antibacterial, anxiolytic, antidepressant, and immunomodulatory properties. It is a rich source of anthocyanins including cyanidin and delphinidin (Abed et al., 2014 ▶). In addition to possessing antioxidant and anti-inflammatory properties, the beneficial cardiovascular properties such as anti-atherosclerotic and antihypertensive effects have been established for anthocyanins (Ojeda et al., 2010 ▶; Garcia-Alonso et al., 2009 ▶). 

Therefore, in the present study, we aimed to evaluate the protective effects of anthocyanin rich extract of *E. amoenum* in human umbilical vein endothelial cells (HUVECs) under oxidative stress induced by H_2_O_2_. The toxicity of *E. amoenum* extract to HUVECs was also studied to confirm the safety of this herbal extract.

## Materials and Methods

**Plant material and preparation of extract**

The petals of *E. amoenum* were collected from around the Qazvin city located 150 km northwest of Tehran, in the Qazvin province during July 2013. A voucher specimen (No. 1147) was deposited at the Herbarium of the School of Pharmacy and Pharmaceutical Sciences, Isfahan, Iran, following identification of the plant by a botanist (Professor Mohammadreza Rahiminejad, Isfahan University, Isfahan, Iran). For the preparation of anthocyanin-rich extract, dried petals of *E. amoenum* were powdered and CH_3_COOH (1%) was added into the materials. The rate of 1:15 was used for *E. amoenum* powder to acetic acid. Using a magnetic blender, the process of extraction continued for 2 h at room temperature. The extract was filtered and treated with dichloromethane (500 mL) three times. Each time, the colored lower phase containing anthocyanins was separated by decanter and treated with ethyl acetate (500 mL) twice. The remaining solution was freeze dried and stored at -20 °C (Gulcin et al., 2005 ▶).

**Determination of total anthocyanin content**

Total anthocyanin content was assessed using the pH differential method. Experiments were carried out in three replicates and data were expressed as cyanidin-3-glucoside equivalent. Briefly, two samples of 0.1 g *E. amoenum* dried extract were mixed with 10 ml of buffer, pH=1 (125 ml of 0.2 M KCl and 375 ml of 0.2 M HCl) as solution A and 10 ml of buffer solution, pH= 4.5 (400 ml of 1 M sodium acetate, 240 ml of 1 M HCl and 360 ml of water) as solution B. Then, the absorbance of both solutions A and B were read at 510 nm. Total anthocyanin amount was determined by the following equation:

Anthocyanin content (%) = [(Abs of solution A – Abs of solution B) × 449.2 × DF / (26900 × Wt)]

Where 449.2 is the molecular mass of cyanidin-3-glucoside chloride and 26900 is molar absorptivity (ε) at 510 nm in the pH= 1, DF is the dilution factor, and Wt is the sample weight (Hasanloo et al., 2011 ▶). 

**Cell culture**

HUVECs were purchased from National Cell Bank of Iran (Pasteur Institute, Iran). They were cultured in a DMEM medium which contained 10% fetal bovine serum (Gibco-I**nvitrogen BioServices Co.,** Bangalore, India), 100 U/ml penicillin, and 100 µg/ml streptomycin and incubated in condition of 5% CO2 and 95% humidified air at 37 °C in 25 and/or 75 cm^2s^ flasks.

**Cell viability evaluation**

For evaluation of the safety of *E. amoenum* extract on HUVECs, cytotoxicity was assessed using 3-(4,5-Dimethylthiazol-2-yl)-2,5-diphenyltetrazolium bromide (MTT) assay (Ma et al., 2011). Briefly, cell suspension at a concentration of 1×10^5^ cells/ml was transferred to 96-well plates. After 24 h incubation at 37 °C, the cells were treated by freshly prepared *E. amoenum* extract at the concentration range of 25 to 1000 µg/ml for an additional 24 h. Afterward, the cells were washed out with phosphate buffered saline (PBS) at PH 7.4, renewed by medium, and 20 μl MTT (0.5 mg/ml; Sigma-Aldrich Co., Madrid, Spain) was added per well and incubated for 3 h in 37 °C. At last, MTT reacted with living cells and converted by mitochondrial enzyme to foramazan crystals with purple color. Foramazan is an insoluble component which is dissolved in dimethyl sulfoxide (DMSO). Its optical density (OD) was measured at 550 nm by microplate reader (BioTek Instruments, PowerWave XS, Wincoski, USA). 

For determination of the protective effect of *E. amoenum* extract on H_2_O_2_-induced oxidative stress, MTT assay was also utilized to determine the survival rate of the HUVECs. After treatment of the HUVECs with freshly prepared *E. amoenum* extract (25-1000 µg/ml) for 24 h at 37 °C, the cells were washed out with PBS. Then, new medium and 0.5 mM hydrogen peroxide (Merck Co., Mumbai, India) were added to the wells and incubated for 2 h. The rest of the experiment was performed as above. 

The wells consisting of cells without being exposed to the extract or H_2_O_2_ were considered as a negative control. DMEM was used as the blank. Cell viability percentage in the negative control was considered as 100. Cell viability was determined through the following formula and each experiment was assayed in triplicate (Sadeghi-Aliabadi et al., 2010 ▶):

Cell viability (%) = (OD test - OD blank/ OD negative control - OD blank) × 100

**Measurement of extra- and intra-cellular hydroperoxides concentration**

The effects of *E. amoenum* extract on intra- and extra-cellular hydroperoxides levels were measured using the ferrous ion oxidation by xylenol orange reagent (FOX-1) (Wolf, 1994 ▶). The FOX-1 reagent comprising ammonium ferric sulfate was provided in aqueous medium with sorbitol based on the manufacturer’s protocol. After pretreatment of HUVECs with *E. amoenum* extract, the cells were stimulated with H_2_O_2_. Then, the samples of supernatant of the cells or the cell lysates from each well were mixed with reagent and after incubation for 30 min in 37 ºC, the solution absorbance was measured at 540 nm using a microplate reader/spectrophotometer. The hydroperoxides concentration of samples was calculated using a H_2_O_2 _standard curve with multiple concentrations.

**Measurement of cell-free and intra- and extra-cellular ferric reducing antioxidant power**

The effect of *E. amoenum* extract on total antioxidant capacity of the samples was determined by the evaluation of ferric reducing antioxidant power (FRAP) (Benzie and Strain, 1996 ▶). FRAP value was measured by the reduction of ferric-tripyridyltriazine complex to ferrous form by colorimetric method. Briefly, the FRAP reagent consisting of tripyridyltriazine/ferric chloride/acetate buffer was provided based on the manufacturer’s protocol and mixed with samples. In cell-free assay, the samples were different concentrations of *E. amoenum* extract in water. In this assay, the samples were supernatant of the cells or the cell lysates from each well. The mixture was incubated for 40 min in 37 °C and then the absorbance of colored solutions was evaluated at 570 nm using a microplate reader/spectrophotometer. The FRAP values of samples were calculated by means of a standard curve acquired from different concentrations of FeSO_4_x7H_2_O and demonstrated by micromole of FeII equivalents per liter.

**Statistical analysis**

All values were represented as the mean±SEM. For statistical examination, a one-way analysis of variance (ANOVA) followed by Tukey’s post-hoc test was used (SPSS software version 16.0). P values0*.*05 were taken as significant.

## Results

**Total anthocyanin content**

Using the pH differential method for dried *E. amoenum* extract, its anthocyanin content equivalent to cyanidin-3-glucoside was detected as 2.5±0.1% (w/w).

**Effect of** ***E. amoenum*** **extract on HUVECs proliferation**

**Figure 1:**
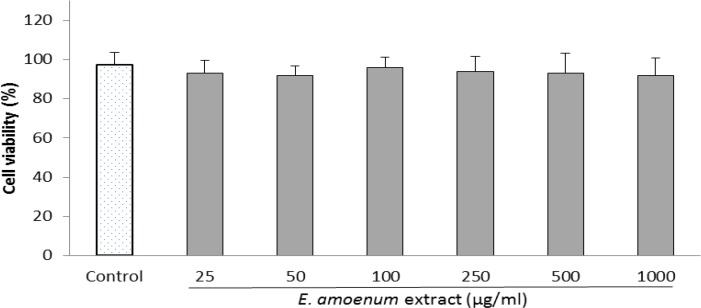
Effect of E. *amoenum* extract on proliferation of HUVECs. Cells were incubated with different concentrations of *E. amoenum* extract (25-1000 μg/ml) for 24 h. The cell viability was determined compared to the control (untreated cells) using the MTT assay. Values are means+SEM from three independent experiments in triplicate.

In order to determine whether *E. amoenum* has cytotoxicity, the effect of *E. amoenum* extract to HUVECs proliferation was evaluated using MTT assay. Non-significant inhibitory effects of *E. amoenum* extract alone (25-1000 µg/ml) for 24 h on HUVECs is shown in Figure 1.

**Cytoprotective effect of** ***E. amoenum*** **extract against H**_2_**O**_2_**-induced oxidative stress **

**Figure 2:**
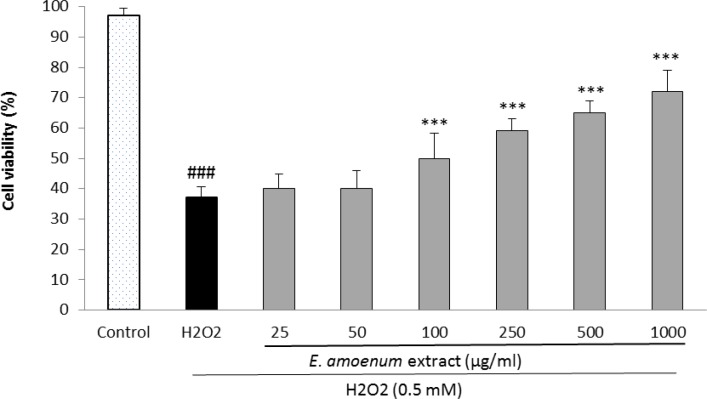
Effect of E. *amoenum* extract on H2O2-induced oxidative stress in HUVECs. Cells were incubated with H_2_O_2_ (0.5 mM, 2h) after pretreatment with different concentrations of *E. amoenum* extract (25-1000 μg/ml). The cell viability was determined using the MTT assay. Values are means+SEM from three independent experiments in triplicate. ###p<0.001 versus control (untreated cells), ***p<0.001 versus H_2_O_2_ stimulated cells.

Figure 2 shows the cytoprotective effect of *E. amoenum* against oxidative stress-induced cell injury on HUVECs using the MTT method. The exposure of HUVECs to 0.5 mM H_2_O_2_ for 2 h resulted in a significant reduction in cell viability (p<0.001).

Pretreatment of HUVECs with *E. amoenum* extract at the concentrations of 100-1000 µg/ml decreased the cell death resulted from the exposure to hydrogen peroxide in a concentration-dependent manner. The protective effect was not observed at the concentrations of 25 and 50 µg/ml of the extract.

**Effects of** ***E. amoenum*** **extract on intra- and extra-cellular hydroperoxides concentration**

**Figure 3:**
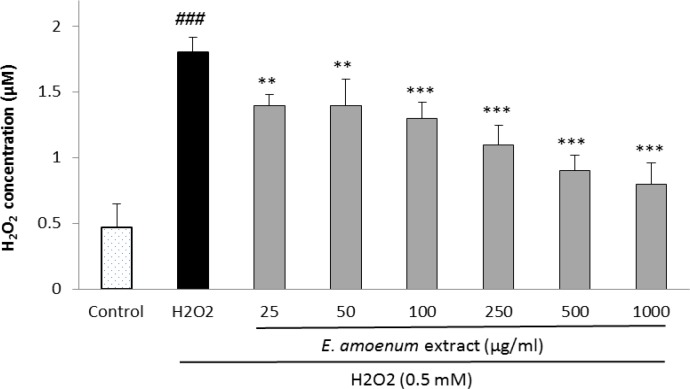
Effect of *E. amoenum* extract on intra-cellular hydroperoxides concentration in HUVECs. Cells were incubated with H_2_O_2_ (0.5 mM, 2h) after pretreatment with different concentrations of E. amoenum extract (25-1000 μg/ml). The hydroperoxides concentration was determined using FOX-1 method. Values are means+SEM from three independent experiments in triplicate. ###p<0.001 versus control (untreated cells), **p<0.01 and ***p<0.001 versus H_2_O_2_ stimulated cells.

**Figure 4:**
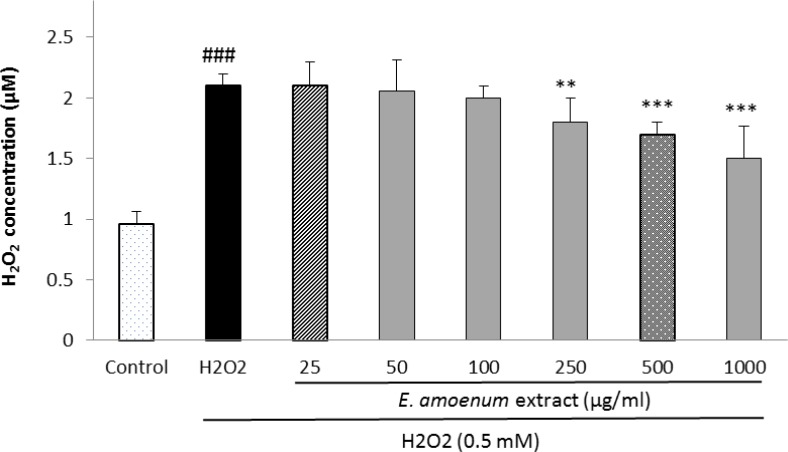
Effect of *E. amoenum* extract on extra-cellular hydroperoxides concentration in HUVECs. Cells were incubated with H_2_O_2_ (0.5 mM, 2h) after pretreatment with different concentrations of E. amoenum extract (25-1000 μg/ml). The hydroperoxides concentration was determined using FOX-1 method. Values are means+SEM from three independent experiments in triplicate. ###p<0.001 versus control (untreated cells), **p<0.01 and ***p<0.001 versus H_2_O_2_ stimulated cells.

The effects of *E. amoenum* extract on intra- and extra-cellular hydroperoxides concentration are shown in Figures 3 and 4. The intra-cellular hydroperoxides levels were significantly reduced after pretreatment of the cells with *E. amoenum* extract at the concentrations of 25-1000 µg/ml compared to the control group***.*** *E. amoenum* extract pretreatment also significantly decreased the extra-cellular hydroperoxides level at the concentrations of 250-1000 µg/ml***.*** Increasing the concentration of the extract significantly prevented the rise in hydroperoxides level concentration-dependently***.***

**Effects of** ***E. amoenum*** **extract on cell-free and intra- and extra-cellular FRAP value **

**Figure 5:**
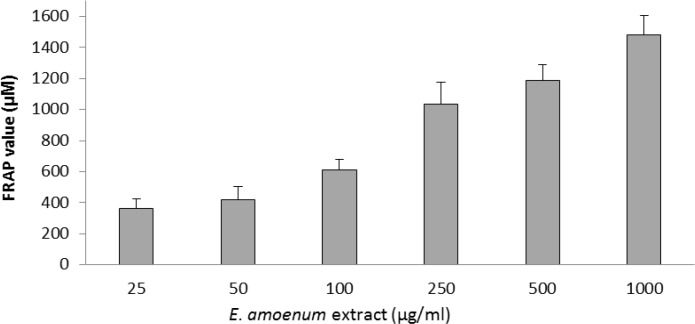
Concentration-dependent FRAP values of different concentrations of *E. amoenum* extract (25-1000 μg/ml). Values are means+SEM from three independent experiments in triplicate.

The FRAP value of the extract of *E. amoenum* petals was expressed as equivalence of ferrous sulphate and our results depicted increasing trend in FRAP with the extract concentrations in cell-free assay (Figure 5).

**Figure 6:**
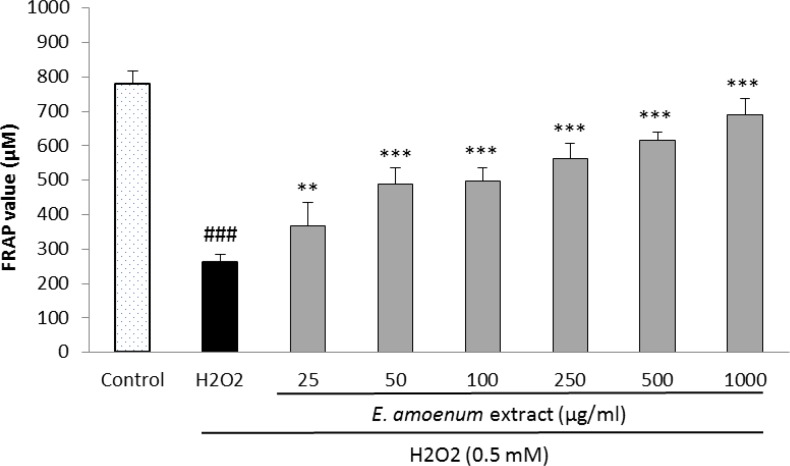
Effect of *E. amoenum* extract on intra-cellular FRAP value in HUVECs. Cells were incubated with H_2_O_2_ (0.5 mM, 2h) after pretreatment with different concentrations of E. amoenum extract (25-1000 μg/ml). Values are means+SEM from three independent experiments in triplicate. ###p<0.001 versus control (untreated cells), **p<0.01 and ***p<0.001 versus H_2_O_2_ stimulated cells.

**Figure 7:**
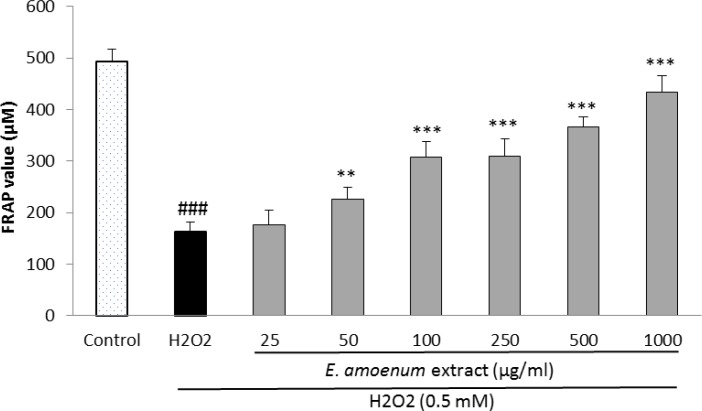
Effect of E. amoenum extract on extra-cellular FRAP value in HUVECs. Cells were incubated with H_2_O_2_ (0.5 mM, 2h) after pretreatment with different concentrations of E. amoenum extract (25-1000 μg/ml). Values are means+SEM from three independent experiments in triplicate. ###p<0.001 versus control (untreated cells), **p<0.01 and ***p<0.001 versus H_2_O_2_ stimulated cells.

In cell-based assay, the FRAP levels were significantly increased at the extract concentrations of 25-1000 µg/ml in intra-cellular fluid (Figure 6) and at the concentrations of 50-1000 µg/ml in extra-cellular fluid (Figure 7), concentration-dependently.

## Discussion

In this study, our findings showed cytoprotective effect of *E. amoenum* extract against oxidative stress induced by H_2_O_2_ in HUVECs at the concentration range of 100-1000 µg/ml. Moreover, it did not show any cytotoxic effects at the concentration range of 25-1000 µg/ml. *E. amoenum* extract also reduced hydroperoxides concentration and increased FRAP value in both intra- and extra-cellular fluid at different concentration ranges. 

For determining the hydroperoxides level, the FOX-1 method was used in this research. FOX assay is a suitable method for detecting hydroperoxides (ROOH) and is not specific only for H_2_O_2_ measurement. This method has two versions including FOX-1 and FOX-2 based on the reagent mediums. It is noteworthy that FOX-1 is more sensitive than FOX-2 for estimation of ROOH (Banerjee et al., 2003 ▶).

In this research, FRAP value was also determined as a measure of total antioxidant capacity of the samples. The antioxidant defense is a multifactorial system which minimizes the tissue damage induced by oxidative stress through preventing ROS generation, destroying potential oxidants, and scavenging ROS. In this system, different antioxidant enzymes including glutathione peroxidase, catalase, and superoxide dismutase remove the potential oxidants (Higashi, et al., 2009 ▶; Benzie and Strain, 1996 ▶). 

Several studies have evaluated beneficial effects of anthocyanins in human populations. Epidemiological studies have demonstrated that anthocyanins reduce the mortality of CVD (Wallace, 2011 ▶). Furthermore, the results of 16 cohort studies proved that the enhancement of flavonoid consumption could reduce the age-related mortality from coronary heart diseases (CHD) (Hertog et al., 1995 ▶). A 16-year follow-up study on postmenopausal women revealed that consumption of some subclasses of flavonoids, including anthocyanins, flavonones, and flavonoid-rich foods resulted in decreased death mortality from CVD and CHD (Mink et al., 2007 ▶). In a randomized double-blind placebo-controlled study, anthocyanins increased high density lipoprotein (HDL) level in pre-hypertensive patients. Therefore, it was concluded that long term consumption of anthocyanins can prevent CVD development (Hassellund et al., 2013 ▶). 

Anthocyanins belong to the flavonoid group of polyphenols with health-promoting properties. These compounds may be beneficial in reducing inflammation and exerting cardiovascular protection. Anthocyanins might be taken up into the vascular endothelial cells. In a study, it has been shown that elderberry anthocyanins were incorporated within the membrane and cytosol and led to the increase of endothelial cell resistance to the damages caused by ROS (Youdim et al., 2000 ▶). Additionally, cyanidin-3-glucoside, the most abundant anthocyanin present in elderberry as in* E. amoenum*, has demonstrated protective effects in vivo. It rapidly enters the cell through bilitranslocase-mediated transport and decreases free radical generation and Cu-induced susceptibility of serum to lipid peroxidation (Khoo et al., 2013 ▶; Ziberna et al., 2012 ▶). 

Anthocyanins have significant antioxidant properties. Cyanidin-3-glucoside has shown to possess stronger antioxidant properties in comparison with other anthocyanins. The cyaniding-3-glucoside-rich extract of defatted dabai peel was also revealed to have potential cardioprotective effects (Tsuda et al., 1998 ▶).

Anthocyanins and their derivatives inhibit platelet activity and aggregation, which can prevent the promotion of cardiovascular disease (Rechner and Kroner, 2005 ▶). They can also influence all of the cells involved in atherosclerosis development. Monocyte chemotactic protein 1 (MCP-1) is a chemokine which plays a direct role in atherogenesis. Red wine anthocyanins have a protective effect against MCP-1 secretion induced by tumor necrosis factor-α (TNF-α) (Garcia-Alonso et al., 2009 ▶). Vascular endothelial growth factor (VEGF) is a pro-atherosclerotic factor. Delphinidin and cyanidin, as the most abundant anthocyanins present in *E. amoenum, *have been able to inhibit VEGF expression which is stimulated by platelet derived growth factor (PDGF) in vascular smooth muscle cells (Oak et al., 2006 ▶). In one study, anthocyanins extracted from chokeberry, bilberry, and elderberry developed vasorelaxation capacity of coronary arteries (Bell and Gochenaur, 2006 ▶). Long term consumption of anthocyanins has resulted in the resistance of the heart to myocardial infarction through elevation of glutathione levels and modulation of cardiac antioxidant defense (Toufektsian et al., 2008 ▶). In an ischemia-reperfusion study, anthocyanins reduced microvascular impairments with preservation of the endothelium and improvement of capillary perfusion (Bertuglia et al., 1995 ▶).

It has also been shown that the anthocyanin delphinidin accelerates the healing of ischemia-reperfusion injury by minimizing the infarct size after ischemia and improvement of the necrosis and apoptosis of cardiomyocytes through inhibition of STAT1 (Scarabelli et al., 2009 ▶).

In summary, the results of this study showed antioxidant and protective effect of anthocyanin-rich extract of the petals of *E. amoenum* against H_2_O_2_-induced oxidative stress in HUVECs. Further investigations are needed for the determination of the mechanisms by which *E. amoenum* exerts its protective effect and understanding its clinical value in human endothelial function.
